# The impact of hypoxia exposure on glucose homeostasis in metabolically compromised humans: A systematic review

**DOI:** 10.1007/s11154-021-09654-0

**Published:** 2021-04-14

**Authors:** Veerle van Hulten, Rens L. J. van Meijel, Gijs H. Goossens

**Affiliations:** 1 Department of Human Biology, NUTRIM School of Nutrition and Translational Research in Metabolism, Maastricht University Medical Center, Maastricht, The Netherlands; 2 Department of Clinical Pharmacology and Toxicology, CARIM School for Cardiovascular Diseases, Maastricht University Medical Center, Maastricht, The Netherlands

**Keywords:** Oxygen, Hypoxia, Glucose homeostasis, Insulin sensitivity, Humans, Randomized controlled trial

## Abstract

Humans living at a higher altitude are less prone to suffer from impaired glucose homeostasis and type 2 diabetes mellitus (T2DM), which might at least partly be explained by lower oxygen availability at higher altitudes. The present systematic review aimed to provide an overview of the current literature on the effects of hypoxia exposure on glucose homeostasis in metabolically compromised humans. Several databases were searched up to August 10^th^, 2020. The search strategy identified 368 unique records. Following assessment for eligibility based on the selection criteria, 16 studies were included in this review. Six studies (2 controlled studies; 4 uncontrolled studies) demonstrated beneficial effects of hypoxia exposure on glucose homeostasis, while 10 studies (8 controlled studies; 2 uncontrolled studies) reported no improvement in glucose homeostasis following hypoxia exposure. Notably, passive hypoxia exposure seemed to improve glucose homeostasis, whereas hypoxic exercise training (2–8 weeks) appeared to have no additional/synergistic effects on glucose homeostasis compared to normoxia exposure. Due to the heterogeneity in study populations and intervention duration (acute studies / 2–8 wks training), it is difficult to indicate which factors may explain conflicting study outcomes. Moreover, these results should be interpreted with some caution, as several studies did not include a control group. Taken together, hypoxia exposure under resting and exercise conditions might provide a novel therapeutic strategy to improve glucose homeostasis in metabolically compromised individuals, but more randomized controlled trials are warranted before strong conclusions on the effects of hypoxia exposure on glucose homeostasis can be drawn.

## Introduction

The current obesity epidemic is accompanied by a proportionate increase in the prevalence of several chronic diseases such as cardiovascular diseases and type 2 diabetes mellitus (T2DM) [1]. With the increasing worldwide prevalence of obesity, interventions targeting obesity and its cardiometabolic complications are increasingly warranted. Many of the current interventions rely on lifestyle modifications or pharmacological treatment. Indeed, the majority of studies have demonstrated that adopting a healthy lifestyle can prevent or delay obesity-related complications, including impaired glucose homeostasis as present in patients with T2DM [2–4]. Importantly, however, dietary changes and regular exercise have often proven hard to maintain for individuals [5]. Additionally, pharmacological therapies often come with side effects [6], and large inter-individual differences in the response to these interventions are often apparent [7]. Therefore, alternative or complementary strategies are needed to mitigate the development of metabolic impairments and related chronic diseases, especially in people at high risk of developing metabolic complications.

The Nobel Prize in Physiology or Medicine was awarded to William Kaelin, Jr., Sir Peter Ratcliffe, and Gregg Semenza in 2019 for their discoveries of how cells sense and adapt to oxygen availability. Intriguingly, humans living at a higher altitude are less prone to suffer from metabolic impairments, independent of body mass index (BMI) [8]. It is tempting to postulate that the lower oxygen availability at higher altitudes may at least partly contribute to the lower prevalence of impaired glucose homeostasis (i.e. T2DM) in highlanders compared to sea level residents. Notably, many confounding factors such as dietary habits and the level of physical activity may also play a role in the beneficial effects of residing at high altitude on glucose homeostasis.

It is well established that adipose tissue (AT) dysfunction and impairments in skeletal muscle (SM) metabolism are major contributors to the development of impaired glucose homeostasis and cardiometabolic complications in humans, as extensively reviewed elsewhere [9]. Interestingly, accumulating evidence indicates that oxygen partial pressure (pO_2_) in AT and SM may affect cellular and organ functioning. Indeed, several *in vitro* experiments using adipocytes and myotubes have demonstrated that pO_2_ is causally related to alterations in glucose and lipid metabolism, as recently reviewed [10]. Under hypoxic circumstances, the body is inclined to switch from fat oxidation towards glucose utilization [10]. In line, acute hypoxia exposure (1% versus 21% O_2_) has been found to increase basal glucose uptake in both human and rodent adipocytes [11–13]. Additionally, we have previously demonstrated that prolonged hypoxia exposure (14 days) to physiological hypoxia (5% O_2_) increased basal glucose uptake in differentiated human multipotent adipose-derived stem cells [14]. Moreover, many studies have shown that pO_2_ alters the expression and secretion of pro-inflammatory cytokines in rodents and human adipocytes [10]. Interestingly, we have previously reported that AT pO_2_ was higher in obese compared to lean individuals [15], and was inversely associated with whole-body insulin sensitivity, independently of adiposity, in men and women [16]. In accordance with these findings, diet-induced weight loss in overweight/obese humans decreased in situ AT pO_2_, with parallel improvements in insulin sensitivity [17]. Finally, several studies have shown that hypoxia exposure increases glucose uptake in rodent and human myocytes, as reviewed [10]. Taken together, previous results from both *in vitro* and *in vivo* studies in rodents and humans indicate that tissue pO_2_ is related to glucose homeostasis and/or insulin sensitivity. Therefore, interventions targeting tissue pO_2_ may provide a promising therapeutic avenue to improve glucose homeostasis in humans.

The aim of the present systematic review was to provide an overview of the current literature on the effects of hypoxia exposure on glucose homeostasis in metabolically compromised humans.

## Methods

### Search strategy

Studies that investigated the effects of hypoxia exposure on glycemic control and/or insulin sensitivity were retrieved from the PubMed database and Web of Science.

The following keyword combinations were used in the PubMed database, sorted by ‘Most recent’: (“hypox*”[tiab] OR “hyperox*”[tiab] OR “low oxygen”[tiab] OR “oxygen deficiency”[tiab] OR “decreased oxygen”[tiab]) AND (“glyc* control” OR “insulin sensitivity” OR "Blood Glucose"[Mesh] OR "Glycemic Index"[Mesh] OR “glucose levels” OR “glycated hemoglobin” OR “glyc* variability” OR “hypoglyc*” OR “hyperglyc*” OR “HOMA index”) AND ("Metabolic Syndrome"[Mesh] OR "Insulin Resistance"[Mesh] OR “prediabetes” OR “impaired glucose tolerance” OR “impaired fasting glucose” OR “prediabetic” OR “obese” OR “obesity” OR “overweight”) NOT (“cancer” AND “apnea” AND “osas”). The results were filtered to only include full text original articles about human studies.

Additionally, the following search string was entered into the Web of Science database: TITLE: ((“hypox*” OR “hyperox*” OR “low oxygen” OR “oxygen deficiency” OR “decreased oxygen”)) AND ALL FIELDS: ((“glyc* control” OR “insulin sensitivity” OR "Blood Glucose"[Mesh] OR "Glycemic Index"[Mesh] OR “glucose levels” OR “glycated hemoglobin” OR “glyc* variability” OR “hypoglyc*” OR “hyperglyc*” OR “HOMA index”) AND ("Metabolic Syndrome"[Mesh] OR "Insulin Resistance"[Mesh] OR “prediabetes” OR “impaired glucose tolerance” OR “impaired fasting glucose” OR “prediabetic” OR “obese” OR “obesity” OR “overweight”)) NOT ALL FIELDS: ((“cancer AND apnea AND osas)). A filter was activated so that only original articles were included.

Searches were not limited by article publication date. The literature search was performed on the 10^th^ of August 2020. From the identified articles, the titles and abstracts were assessed and, if considered relevant for the present systematic review, the full text of the article was examined in detail.

### Selection criteria

Eligible studies included humans with impaired glucose homeostasis. Thus, patients with overweight/obesity, the metabolic syndrome and/or patients with T2DM were included. Only studies with a clear description of the intervention (and control condition, if applicable), as well as parameters related to glucose metabolism as study outcomes were included. Only original articles written in English were included.

## Results

### Literature search

**Fig. 1:**
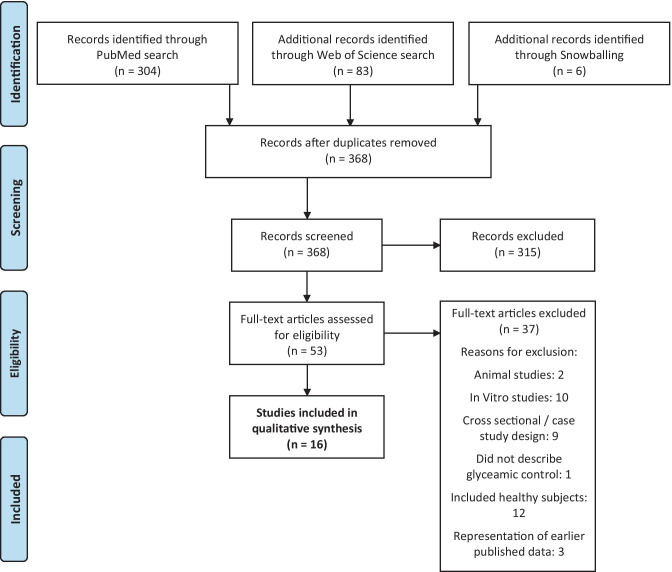
Flow-chart illustrating the results of the database search

A flowchart to illustrate the inclusion process is shown in Fig. 1. The literature search identified 368 unique records. Titles and/or abstracts were screened, which resulted in the exclusion of 315 studies. 6 additional records were identified through searching reference lists. The remaining 53 full-text articles were retrieved and assessed for eligibility based on the selection criteria. From these full-text articles, a total of 37 articles were excluded. Reasons for exclusion were related to the study population, study design and outcome parameters. Thus, a total of 16 studies were included in the present systematic review. From these studies, 10 studies were randomized controlled trials (RCTs), whereas the other 6 studies did not include a control group.

### The effects of hypoxia exposure on glucose homeostasis

As mentioned earlier, previous *in vitro* and *in vivo* studies in rodents have indicated that hypoxia exposure impacts glucose homeostasis, as reviewed recently [10]. Based on these studies, it could be argued that hypoxia exposure may provide a potential therapeutic strategy to combat disturbances in glucose metabolism. However, only a limited number of *in vivo* studies have investigated the effects of hypoxia exposure on glucose homeostasis in metabolically compromised humans. Here, we systematically reviewed the literature to provide an overview of the effects of hypoxia exposure under resting conditions as well as hypoxia exposure during exercise on glucose homeostasis in metabolically compromised individuals.

#### The effects of hypoxia exposure on circulating glucose and insulin concentrations

Several but not all studies have shown beneficial effects of hypoxia exposure on glucose homeostasis (Table 1). Duennwald et al. [18] have found that merely one hour of passive intermittent hypoxia (F_i_O_2_ 13%) exposure significantly decreased fasting plasma glucose levels in individuals with overweight, while normoxia exposure did not significantly change glucose concentrations. Notably, the difference between the effects of hypoxia compared with normoxia exposure was statistically significant [18]. Additionally, Serebrovska et al. [19] found that three weeks of moderate passive intermittent hypoxia exposure (F_i_O_2_ 12%, 40 min/day, 3x/week) improved fasting and postprandial glucose concentrations in healthy individuals as well in as people with impaired glucose homeostasis. In line, Lecoultre et al. [20] reported significantly lower fasting glucose levels in obese individuals after ten consecutive nights of passive moderate hypoxia exposure (~ 10 h/night at F_i_O_2_ ~ 15% O_2_). Importantly, however, the latter two studies did not include a control group [19, 20]. Furthermore, Mackenzie et al. [21] have shown that plasma insulin levels during a 4-h intravenous glucose tolerance test were significantly decreased after one 60-min bout of hypoxic exercise (F_i_O_2_ 14.6%) at 90% of the lactate threshold compared to normoxic exercise at the same relative intensity in T2DM patients. In a follow-up study, Mackenzie et al. [22] demonstrated that exercise for 60 min at 90% of the lactate threshold in hypoxic conditions (F_i_O_2_ ~ 15% O_2_) decreased fasting glucose and insulin levels in T2DM patients, while these effects were not present following only 20 min of hypoxic exercise. Notably, it is not clear whether these beneficial effects were due to hypoxia exposure or stem purely from the exercise bout, as a control group was not included in this study [22]. In addition, Marlatt et al. [23] found a significant reduction in 2-h glucose levels during an OGTT in metabolically compromised individuals following 14 nights of exposure to moderate hypoxia (sleeping at home in a hypoxic tent maintained at 15% O_2_), although fasting glucose levels remained unchanged. Also in the latter study, no control group was included [23]. Finally, De Groote et al. [24] demonstrated that six weeks of hypoxic training (F_i_O_2_ 15%, 3 × 1 h/wk) but not normoxic training significantly decreased plasma insulin levels during an oral glucose tolerance test and improved glucose tolerance in obese adolescents. Yet, the differences between the effects of hypoxic and normoxic training on postprandial glucose and insulin concentrations did not reach statistical significance in the latter study [24].

**Table 1:** Study characteristics and outcomes

Authors	Characteristics of study participants	Hypoxia exposure protocol	Main outcome parameters	Study outcomes*
Duennwald et al. 2013 [18]	T2DM patients (*n *= 14; 3 females); age 59.3 ± 1.5 yrs; BMI 29.4 ± 1.0 kg/m^2^.	Single-blind, placebo-controlled study. 1h intermittent hypoxia (5 x 6 min at F_i_O_2_ 13%, interspersed with 5 x 6 min at F_i_O_2_ 21%) or normoxia (continuous F_i_O_2_ 21%) exposure. Blood glucose concentration was measured at t=0, 3h and 6h following the 1h-exposure. Wash-out period: 3–5 days.	Plasma glucose values (mmol/L)	Δ fasting plasma glucose (mmol/L) (post-baseline): • Hypoxia group: -2.1 * P < 0.01* • Placebo group: -0.8 * P* = *N.S* Hypoxia versus normoxia: * P *= 0.037
Serebrovska et al. 2017 [19]	Individuals with IFG (5.6-6.9 mmol/L) and/or IGT (2h plasma glucose 7.8-11.0 mmol/L) (*n *= 11; 6 females); age 66.4 ± 5.2 yrs; BMI 33.2 ± 5.6 kg/m^2^).	Measurements were performed at baseline, one day (acute normobaric hypoxic test: 12% O_2_ for 20 min) and one month after intermittent hypoxia exposure. Participants wore a mask during hypoxia exposure, 3x/wk for 3 weeks. Each session consisted of 4 x 5-min normobaric hypoxia (F_i_O_2_ 12%) followed by 5-min normoxia (room air breathing).	Fasting and post-glucose ingestion serum glucose (mmol/L) concentration pre and one month post intermittent hypoxic training.	Δ fasting serum glucose (mmol/L) (post-pre): • -0.4 * P < 0.05* Δ 2h OGTT serum glucose concentration (mmol/L) (post-pre): • -1.5 * P < 0.01*
LeCoultre et al. 2013 [20]	Obese men (*n *= 8; 4 Caucasians, 3 African Americans, and 1 Hispanic); age 28 ± 1 yrs; BMI 32.7 ± 1.3 kg/m^2^	Hypoxia exposure (F_i_O_2_ 15%) for 10 consecutive nights (±10 hours/night in hypoxic tent). Insulin sensitivity was measured by a two-step hyperinsulinemic-euglycemic clamp at baseline and on day 12.	Fasting insulin (mU/L), fasting glucose (mg/dL) and glucose disposal rate (mg/kg/min)	Δ fasting glucose (mg/dL) (post-pre): • -3.0 * P < 0.05* Δ glucose disposal rate (mg/kg/min) (post-pre): • 0.9 * P < 0.05* Δ fasting insulin (mU/L) (post-pre): • -0.8 * P* = *N.S.*
Mackenzie et al. 2011 [21]	Sedentary men (*n *= 8) with recently-diagnosed (<5 yrs) T2DM (age 58 ± 4 yrs, BMI 29.2 ± 6.7 kg/m^2^).	Following an overnight fast (∼12 h), subjects completed four trials: 60 min of 1) normoxic rest (F_i_O_2_ 21%), 2) hypoxic rest (F_i_O_2_ 14.6%), 3) normoxic exercise (F_i_O_2_ 21%) and 4) hypoxic exercise (F_i_O_2_ 14.6%). Dietary intake was controlled 48 h prior to each condition. Five subjects were diet treated, and three subjects were treated with metformin. Subjects were asked to abstain from medication 48 h the before experimental trials.	Insulin sensitivity (S_i_^2^*), AUC for arterialized blood glucose (mmol/L/4h) and AUC for plasma insulin (μU/mL/4h) during a 4-h intravenous glucose tolerance test	Δ Insulin sensitivity (S_i_^2^*, ∙10^4^ μU/ml) (normoxic – hypoxic): • Rest: -0.86 * P < 0.05 * • Exercise: -1.13 * P < 0.05* Δ AUC for plasma insulin (μU/mL/4h) (normoxic-hypoxic): • Rest: 1861 * P < 0.05* • Exercise: 1303 * P < 0.01* Δ AUC for arterialized blood glucose (mmol/L/4h) (normoxic-hypoxic): • Rest: 204 * P* = *N.S.* • Exercise: 120 * P* = *N.S.*
Mackenzie et al. 2012 [22]	Sedentary males (*n *= 8), with recently diagnosed T2DM; age 57.5 ± 2.3 yrs, BMI 29.2 ± 2.9 kg/m^2^).	Subjects performed 3 acute exercise sessions (60, 40 and 20 minutes at 90% of predetermined lactate threshold) under hypoxia (F_i_O_2_ 14.7±0.2%) separated by at least 7 days. After each exercise trial (day 1), fasting glucose and HOMA-IR were determined after 24 hours (day 2) and 48 hours (day 3). No normoxic exercise control group was included.	Fasting glucose (mmol/L), fasting insulin (μU/ml), HOMA-IR.	Δ fasting glucose (mmol/L) (day 3-day 1): • 20 minutes: -0.46 * P* = *N.S.* vs day 1 • 40 minutes: -0.87 * P < 0.05* vs day 1 • 60 minutes: -1.01 * P < 0.05* vs day 1 Δ fasting insulin (μU/ml) (day 3-day 1): • 20 minutes: -0.20 * P* = *N.S.* vs day 1 • 40 minutes: -2.40 * P* = *N.S.* vs day 1 • 60 minutes: -2.67 * P < 0.05* vs day 1 Δ HOMA-IR (day 3-day 1): • 20 minutes: -0.18 * P* = *N.S.* vs day 1 • 40 minutes: -1.47 * P < 0.05* vs day 1 • 60 minutes: -1.96 * P < 0.05* vs day 1
Marlatt et al. 2020 [23]	Adults with confirmed T2DM (*n *= 8, 3 females) Age 49 ± 10 yrs; BMI 39.6 ± 5.8 kg/m^2^.	Participants were required to sleep for 14 consecutive nights (7–12 h per night) at home in a hypoxic tent maintained at ~15% O_2_ (range 14.5– 15.5% O_2_). Participants completed inpatient visits at day 0 (baseline, pre-intervention) and day 14 (end of intervention).	2-h plasma glucose AUC (in mg/dL × h), 2- h plasma insulin AUC (in μU/mL × h), and insulin sensitivity as estimated by the Matsuda Index and Disposition Index	Δ 2-h plasma glucose AUC (in mg/dL × h) (post-pre): • -62 * P < 0.05* Δ insulin sensitivity (estimated by Matsuda Index) (post-pre): • 0.5 * P* = *N.S.* Δ insulin sensitivity (estimated by Disposition Index) (post-pre): • 0.5 * P* = *N.S.*
De Groote et al. 2018 [24]	Adolescents (*n *= 14, 8 females), aged 12–15 yrs; BMI >30 kg/m^2^.	8 week, randomized, single-blind study, including 6 weeks of exercise training where three times per week, adolescents were trained for 50– 60 min, including 12 min on a cycle ergometer - Session 1: 2 min at 50% MAP and 10 min at 70% MAP - Session 2: 2 min at 50% MAP and 5 repetitions of 1 min 80%–1 min 50% MAP; - Session 3: incremental training started at 40% MAP with an increase of 10% MAP each 2 min and resistance training of the abdominal, quadriceps, and biceps muscles (15 repetitions at 50% 1RM + 4 sets of 6 repetitions at 70% 1RM; resting time: 2 min). The normoxic exercise group (n = 7, 4 females) was exposed to ambient conditions, while the normobaric hypoxic exercise group (n = 7; 4 females) was exposed to a F_i_O_2_ 15%.	HOMA-IR, HOMA-β, QUICKI, AUC plasma insulin (µU/ml) and glucose (mg/dl).	Δ AUC plasma insulin (%) (post-pre): • Hypoxia group: -49 * P < 0.01* • Normoxia group: -21 * P* = N.S. Δ AUC glucose levels (%) (post-pre): • Hypoxia group: -14 * P < 0.01* • Normoxia group: -7 * P* = N.S. Δ HOMA-IR (%) (post-pre): • Hypoxia group: -37 * P = 0.08* • Normoxia group: -45 * P < 0.05* Δ HOMA-β (%) (post-pre): • Hypoxia group: -30 * P < 0.05* • Normoxia group: -29 * P < 0.05* Δ QUICKI (%) (post-pre) • Hypoxia group: 7 * P < 0.01* Normoxia group: 6 *P < 0.01*
Wiesner et al. 2009 [25]	Sedentary, nondiabetic or insulin resistant, overweight/obese men and women (*n *= 45, 27 females). Age 42 ± 7.1 yrs; BMI: 30.2 ± 3.6 kg/m^2^.	Subjects were submitted to a training program (60 min/day, 3x/week) for 4 weeks at a heart rate corresponding to 65% of maximum oxygen consumption under normobaric normoxia (F_i_O_2_ 21.0%) or hypoxia (F_i_O_2_ 15%). Both groups trained at the same relative exercise intensity.	HOMA-IR, fasting insulin (µU/ml).	Δ HOMA-IR (post-pre): • Hypoxia group: -0.8 * P < 0.05* • Normoxia group: -0.7 * P < 0.05* Δ fasting insulin (µU/ml) (post-pre): • Hypoxia group: -3.1 * P < 0.05* • Normoxia group: -3.2 * P < 0.05*
Lippl et al. 2010 [26]	Obese men with the metabolic syndrome (*n *= 20); Age 55.7 ± 4.1 yrs, BMI 33.7 ± 1.0 kg/m^2^).	Baseline (day 1) and follow-up investigations (day 42) were performed at an altitude of 530m. High-altitude measurements (day 7 and day 14) were performed at an altitude of 2,650m. Activity was restricted to slow walks. The selection and amount of foods was similar at the different locations. Testing was performed at the same time of day for each patient after a minimum fast of 8h.	Fasting glucose (mg/dl), insulin values (μU/ml), HbA1c (%) and HOMA.	Δ HbA1c (%) (day 14-day 1): • -0.1 * P < 0.05* Δ fasting blood glucose (mg/dl) (day 14-day 1): • -1.9 * P* = *N.S.* Δ insulin (μU/ml) (day 14-day 1): • 3.5 * P* = *N.S.* Δ HOMA-IR (day 14-day 1): • 1.3 * P* = *N.S.*
Morishima et al. 2015 [27]	Sedentary men (*n *= 21); Age 24.3 ± 1.12 yrs; BMI 25.5 ± 0.7 kg/m^2^).	Subjects were randomly assigned to either the 2-week hypoxic training group (n = 11; 6 sessions/week) or the 4-week hypoxic training group (n = 10; 3 sessions/week). Each training session consisted of 60-min cycling at 65% of maximal oxygen uptake (VO_2max_) evaluated under hypoxic conditions (F_i_O_2_ 15.0%).	Fasting glucose (mg/dl) and insulin values (μU/ml)	Δ fasting glucose (mg/dl) (post-pre): • 2-week group: -1.0 * P* = *N.S.* • 4-week group: -1.0 * P* = *N.S.* Δ fasting insulin (μU/ml) (post-pre): • 2-week group: -2.0 * P* = *N.S.* • 4-week group: -2.5 * P* = *N.S.*
Morishima et al. 2014 [28]	Sedentary, overweight men (*n *= 8); Age 27 ± 3 yrs; BMI 28.6 ± 0.8 kg/m^2^).	Maximal oxygen uptake (VO_2max_) was assessed using a graded power test on an ergometer under normoxic (first visit) and hypoxic (second visit) conditions. Four acute experimental studies were carried out in a randomized crossover design (sessions separated by ± 7 days) in an environmental chamber following an overnight fast: both a 1) rest and 2) exercise trial under normoxic conditions (F_i_O_2_ = 20.9%), and a 3) rest and 4) exercise trial under hypoxic conditions (F_i_O_2_ = 15.0%). In the rest trials subjects rested on a chair. In the exercise trials, subjects conducted 3x30 min pedaling exercise at 60% of VO_2max_ at 8:00, 10:30, and 13:00, and rested during the remaining periods. Standard meals were provided at 8:30, 11:00, and 13:30.	AUC for glucose (mg/dL ∙7.5h) and AUC for serum insulin (μIU/∙7.5h), including intake of meals with or without exercise sessions.	Δ AUC for blood glucose (mg/dL∙7.5h ) (normoxic-hypoxic) (data not shown in text but in figures): • Rest : * P* = *N.S.* • Exercise: * P* = *N.S.* Δ AUC for serum insulin (μIU/mL∙7.5h ) (normoxic-hypoxic): • Rest: *P* = *N.S.* • Exercise: *P* = *N.S.*
Gutwenger et al. 2015 [29]	Individuals with the metabolic syndrome (*n *= 14; 8 females); high altitude: Age: 39 – 60 yrs; BMI 31.1 ± 5.3 kg/m^2^ Low altitude: Age 55 – 69 yrs; BMI 32.3 ±4.2 kg/m^2^.	Participants were assigned to two groups: exercise under mild hypobaric hypoxic conditions at 1,900 m altitude for 2 weeks (n = 8), or exercise under normobaric normoxic conditions at 300 m altitude (n = 6) for 2 weeks. Both groups participated in the supervised training program (hiking; 3h/day, 4x/week; total training time of 24 hours) at an intensity of 55–65% of the individual maximal heart rate.	Insulin values (mU/L) and glucose values (mg/dl).	Δ blood insulin values (mU/L) (post-pre): • Moderate altitude: 1.0 • Low altitude: 1.5 Moderate versus low altitude: * P* = *N.S.* Δ blood glucose values (mg/dl) (post-pre): • Moderate altitude: -1.6 • Low altitude: -1.5 Moderate versus low altitude: * P* = *N.S.*
Chacaroun et al. 2020 [30]	Overweight or obese, sedentary subjects (*n *= 23, 4 females); Age 54 ± 11 yrs; BMI 31.5 ± 2.8 kg/m^2^).	Subjects performed 3x45-min exercise sessions/week for 8 weeks. Measurements were performed before and after the 8-week training period. During cycling, workload was continuously adjusted to obtain a heart rate of 75% of individual maximal heart rate. For the hypoxic exercise training group (11 males and 1 female), F_i_O_2_ was individually and continuously adjusted to reach a target SpO_2_ of 80% under normobaric conditions. The normoxic exercise training group (8 males and 3 females) inhaled normoxic ambient air (F_i_O_2_ 21%).	HOMA2-IR, plasma insulin (μU/ml).	Δ HOMA2-IR (post-pre): • Hypoxia group: -0.03 * P* = *N.S.* • Normoxia group: -0.05 * P* = *N.S.* Δ plasma insulin (μU/ml) (post-pre): • Hypoxia group: -0.4 *P* = *N.S.* • Normoxia group: -0.4 *P* = *N.S.*
Klug et al. 2018 [31]	Men with metabolic syndrome (*n *= 23); Age 18-70 yrs; BMI hypoxic group 34.1±0.9, BMI normoxic group 35.5±1.4 kg/m^2^.	Patients completed a 6-week moderate, aerobic exercise program (3x/wk at 50–60% of maximal heart rate). One training session lasted 60 min with 3x15 min intervals of walking on the treadmill and a 5 min break in between for recovery. The hypoxic exercise group was exposed to normobaric hypoxia (F_i_O_2_ 15%), while the normoxic exercise group was exposed to normobaric normoxia (F_i_O_2_ 21%)	Glucose (mmol/L) and insulin levels (μU/ml), HbA1c (%).	Δ fasting insulin (μU/ml) ( post-pre): • Hypoxia group: 3 * P* = *N.S.* • Normoxia group: -2 * P* = *N.S.* Hypoxia versus normoxia: * P *= *N.S.* Δ blood glucose (mmol/L) (post-pre): • Hypoxia group: -0.2 * P* = *N.S.* • Normoxia group: 0 * P* = *N.S.* Hypoxia versus normoxia: * P *= *N.S.* Δ HbA1c (%) (post-pre): • Hypoxia group: -0.2 * P* = *N.S.* • Normoxia group: -0.1 * P* = *N.S.* Hypoxia versus normoxia: * P *= *N.S.*
Chobanyan-Jürgens et al. 2019 [32]	Older sedentary individuals (*n *= 29, 14 females), Hypoxic group: Age 60.4±5.1 yrs; BMI 28.6±3.0 kg/m^2^. Normoxic group: Age 63.8±5.8 yrs; BMI 28.3±1.9 kg/m^2^.	Participants trained on a bicycle ergometer 3 days per week for 8 weeks under normobaric hypoxia (F_i_O_2_ 15%) or normoxia. Subjects trained at a heart rate corresponding to 60% of pre-training VO_2_-peak for 30 minutes for the first 4 weeks. After 4 weeks, exercise intensity was increased to 70% of pre-training VO_2_-peak and exercise duration to 40 minutes. Pre-training VO_2_-peak was determined by performing an incremental exercise test on a bicycle ergometer.	Glucose infusion rate (mg/min), HOMA-IR and the insulin sensitivity index (μg/kg/min/(mM∙pM))	Δ glucose infusion rate (mg/min) (post-pre): • Hypoxia group: 74 * P < 0.01* • Normoxia group: 54 * P < 0.05* Hypoxia versus normoxia: * P *= *N.S.* Δ HOMA-IR (post-pre): • Hypoxia group: -0.5 * P* = *N.S.* • Normoxia group: -0.2 * P* = *N.S.* Hypoxia versus normoxia: * P *= *N.S.* Δ insulin sensitivity index ((μg/kg/min/(mM∙pM)) (post-pre): • Hypoxia group: 0.17 * P < 0.05* • Normoxia group: 0.16 * P < 0.05* Hypoxia versus normoxia: * P *= *N.S.*
Shin et al. 2018 [33]	Japanese men (*n *= 41), Hypoxic group: Age 45.6±20.9 yrs; BMI 26.8±2.3 kg/m^2^. Normoxic group: Age 46±20.5 yrs; BMI 27±3 kg/m^2^.	Subjects trained on a treadmill 3 days per week for 4 weeks, for 50 min (including 5-minute warm-up and cool-down periods). A 30-minute rest period preceded and followed each exercise session. Exercise was performed at an intensity corresponding to 60% of the maximum heart rate, which was calculated from the age and heart rate at rest. Exercise was performed at either normobaric hypoxic (15.4% O_2_) conditions (equivalent to an altitude of 2500 m), or normobaric normoxic (20.9% O_2_) conditions (equivalent to sea level).	HOMA-IR, fasting insulin (μU/ml) and glucose (mmol/L).	Δ HOMA-IR (post-pre): • Hypoxia group: -1.13 * P < 0.05* • Normoxia group: -0.22 * P < 0.05* Δ fasting insulin (μU/ml) (post-pre): • Hypoxia group: -5.43 *P* = *N.S.* • Normoxia group: -0.85 *P* = *N.S.* Δ blood glucose (mmol/L) (post-pre): • Hypoxia group: -3.25 * P* = *N.S.* • Normoxia group: -4.14 * P < 0.01*

*1RM *one repetition maximum, *AUC *area under the curve, *BMI *body mass index, *F*_i_*O*_2_ fraction of inspired oxygen, *HbA1c *glycated hemoglobin, *HOMA-β *homeostatic model assessment for β-cell function, *HOMA-IR *homeostatic model assessment for insulin resistance, *IFG *impaired fasting glucose, *IGT *impaired glucose tolerance, *MAP *mean arterial pressure, *N.S. *nonsignificant, *OGTT *oral glucose tolerance test, *QUICKI *quantitative insulin sensitivity check index, *T2DM *type 2 diabetes, *VO*_2_*max* maximal oxygen uptake

* ‘Pre-post’: The difference from baseline to post-intervention (pre-intervention values minus post-intervention values)

Taken together, these studies suggest that passive hypoxia exposure (i.e. hypoxia exposure under resting conditions) [18–20] as well as hypoxic exposure during an acute bout of exercise [18, 22] or exercise training program [24] may improve glucose homeostasis in metabolically compromised individuals.

In contrast, several other studies that investigated the effects of hypoxic exercise on glucose homeostasis did not find significant changes in parameters related to glucose homeostasis (Table 1). Wiesner et al. [25] have shown that a four-week hypoxic training program (F_i_O_2_ ~ 13% O_2_, 60 min/day, 3 days/week) at a heart rate corresponding to 65% of maximum oxygen consumption significantly decreased fasting insulin levels compared to baseline in metabolically compromised individuals. Importantly, however, an identical training program under normoxic circumstances yielded similar effects, suggesting that these improvements are likely due to exercise per se rather than hypoxia exposure. Lippl et al. [26] found that seven days of hypobaric hypoxia exposure (altitude of 2,650 m) did not alter metabolic parameters, including fasting glucose and insulin levels, compared with normobaric normoxia exposure (altitude of 530 m) in metabolically compromised individuals. Somewhat surprising, however, a significant decrease in HbA1c levels following one week of hypoxic exposure was found in the latter study, which was not yet seen after seven days of normoxic exposure [26]. Similarly, Morishima et al. [27, 28] demonstrated that hypoxia exposure did not significantly impact fasting and postprandial glucose concentrations in overweight men, despite a pronounced increase in postprandial carbohydrate oxidation under hypoxic resting and exercise conditions compared to normoxia exposure [28]. Neither hypoxia exposure (F_i_O_2_ 15%) under resting conditions [28], nor hypoxic exercise (12 × 1 h training sessions over 2–4 wks) [27] altered fasting blood glucose and serum insulin concentrations. Furthermore, Gutwenger et al. [29] have shown that fasting plasma glucose and insulin levels were not significantly affected in response to a two-week hiking vacation (3 h/day, 4 days/week) at high (1,900 m) versus low (300 m) altitude in patients with the metabolic syndrome. Likewise, Chacaroun et al. [30] were not able to show any improvements in plasma insulin levels and HOMA2-IR after an eight-week hypoxic exercise intervention with target SpO_2_ of 80% during the exercise sessions in overweight and obese individuals. Finally, Klug et al. [31] found that a six-week hypoxic (F_i_O_2_ 15%) exercise intervention in men with the metabolic syndrome did not significantly change blood glucose and insulin levels after an oral glucose load. In line, no differences were found in glucose tolerance and HbA1c between normobaric hypoxic and normobaric normoxic training conditions [31].

Thus, several studies showed no additional benefits of an acute bout of exercise [28] or hypoxic exercise training for 2–8 weeks [25, 27, 29–31] on glucose homeostasis in metabolically compromised individuals.

To summarize, 6 studies (2 controlled studies; 4 uncontrolled studies) demonstrated beneficial effects of hypoxia exposure on glucose homeostasis, while 10 studies (8 controlled studies; 2 uncontrolled studies) reported no improvements in glucose homeostasis following hypoxia exposure. Notably, passive hypoxia exposure seemed to improve glucose homeostasis, while hypoxic exercise training (2–8 weeks) appeared to have no additional/synergistic effects on glucose homeostasis compared to normoxia exposure.

#### The effects of hypoxia exposure on insulin sensitivity

Although conflicting findings on the effects of hypoxia exposure on circulating glucose and insulin concentrations have been reported (Table 1), results of studies that have examined the effects of hypoxia exposure using more sophisticated measures of insulin sensitivity and glucose tolerance such as the hyperinsulinemic-euglycemic clamp and the intravenous glucose tolerance test, respectively, suggest that hypoxia may improve insulin sensitivity.

Lecoultre et al. [20] showed that ten consecutive nights of moderate hypoxia (F_i_O_2_ 15%) exposure improved whole-body insulin sensitivity, assessed with the gold-standard hyperinsulinemic-euglycemic clamp. Strikingly, the improvements in whole-body insulin sensitivity were more pronounced in individuals with the lowest baseline insulin sensitivity, underlining the potential of hypoxia exposure as a therapy for people with (severe) insulin resistance. Yet, the latter study did not include a control group [20]. Furthermore, Mackenzie et al. [21] demonstrated improvements in glucose tolerance, determined using an intravenous glucose tolerance test, in response to acute hypoxia (F_i_O_2_ 15%) compared to normoxia exposure in patients with T2DM. Conversely, a study performed by Chobanyan-Jürgens et al. [32] did not show any significant additive beneficial effects of exercise under hypoxic circumstances (F_i_O_2_ 15%, 3 × 30-40 min/week for 8 weeks) compared to normoxic circumstances on insulin sensitivity, assessed by a hyperinsulinemic-euglycemic clamp, in older sedentary overweight individuals.

In addition, studies that used surrogate markers of insulin sensitivity found improved [21, 22, 24, 25, 32, 33] or unchanged [27–31] insulin sensitivity following a hypoxia exercise program. Importantly, several of the studies that did find improved insulin sensitivity following hypoxia exposure did not include a control group or the improvement in the active arm (hypoxia exposure) was not different from the control condition (normoxia exposure) [24, 25, 32].

Thus, both acute and more prolonged exposure to hypoxia with or without the addition of exercise may have beneficial effects on insulin sensitivity in metabolically compromised individuals, but conflicting findings have been reported.

## Discussion

The findings of the studies included in the present systematic review suggest that hypoxia exposure has beneficial or neutral effects on glucose homeostasis in metabolically compromised humans. More specific, passive hypoxia exposure seemed to improve glucose homeostasis, whereas hypoxic exercise training (2–8 weeks) appeared to have no additional/synergistic effects on glucose homeostasis compared to normoxia exposure. Due to the heterogeneity in study populations in these studies with respect to age, sex, metabolic status (i.e. no T2DM/T2DM) and ethnicity, as well as differences in severity and duration (acute studies / 2–8 wks training) of hypoxia exposure, it is difficult to indicate which factors may explain the different study outcomes. Importantly, as several studies that reported beneficial effects on glucose homeostasis did not include a control group, these results should be interpreted with caution. Clearly, more well-controlled studies are needed to be able to disentangle which parameters related to study population, hypoxia exposure protocol and/or intervention duration determine intervention outcomes.

Human studies have demonstrated a great variety in responses to hypoxia exposure on glucose homeostasis. One could argue that exercise under mild hypoxic conditions might induce more pronounced adaptations in glucose homeostasis as compared to hypoxia exposure under resting conditions due to a stronger hypoxic stimulus under exercise conditions [34]. Even more importantly, the adaptations underlying the effects of hypoxia may vary based on the severity and duration of hypoxia exposure (duration of each hypoxic episode and total exposure duration) as well as the pattern (intermittent/continuous) of exposure [10]. Indeed, it has been suggested that mild hypoxia exposure with low cycle numbers (3–15 cycles/day) may improve several metabolic parameters, whereas severe hypoxia, with high-frequent episodes may result in pathological adaptations [35]. For example, severe (intermittent) hypoxia exposure may increase sympathetic nervous system activity, blood pressure, inflammation, cholesterol levels, the risk of atherosclerosis and right ventricular hypertrophy, and impair cognitive function, as extensively reviewed [35]. Since severe hypoxia exposure may induce Acute Mountain Sickness symptoms and other pathogenic effects, it is important to closely monitor adverse events when conducting hypoxia exposure intervention studies. Importantly, none of the studies included in the present systematic review reported more Adverse Events related to the intervention during hypoxia exposure compared to normoxia exposure. The results of the studies included in the present systematic review seem conflicting and are difficult to compare due to the heterogeneity of study designs. In addition, several studies did not include a control group, which clearly hampers robust conclusions on the effects of hypoxia exposure on glucose homeostasis and insulin sensitivity.

At the cellular level, findings on the effects of hypoxia exposure on glucose homeostasis are less conflicting. A potential mechanism by which hypoxia causes a decrease in blood glucose levels is by inducing a switch from aerobic to anaerobic metabolism, mediated by the hypoxia-inducible factor (HIF)-1 system [10, 36]. Indeed, both hypoxia exposure and contraction (*in vitro* electrical pulse stimulation) have been demonstrated to improve insulin action and glucose metabolism in myotubes via activations of the HIF-1α pathway [37]. In addition, hypoxia and exercise induced an increase in the AMP/ATP ratio in soleus muscle of lean rats, thereby increasing insulin-independent glucose uptake through AMP-activated protein kinase activity [38]. Moreover, hypoxia increased the intracellular free Ca^2+^ levels and subsequently calmodulin-dependent protein kinase, ultimately enhancing insulin-independent glucose uptake [39]. Interestingly, synergistic effects of hypoxia exposure and contraction have been demonstrated regarding stimulation of glucose transport in rat hindlimb muscle [40]. Furthermore, many experiments have demonstrated that changes in oxygen levels impact the functionality of (pre)adipocytes and immune cells, leading to alterations in adipose tissue inflammation, lipid and glucose metabolism, as extensively reviewed elsewhere [10].

In conclusion, the results of the studies included in the present systematic review suggest that hypoxia exposure, either under resting conditions or during exercise, might provide a novel, non-pharmacological therapeutic strategy to improve glucose homeostasis in metabolically compromised individuals. More specific, passive hypoxia exposure seemed to improve glucose homeostasis, whereas hypoxic exercise training (2–8 weeks) appeared to have no additional/synergistic effects on glucose homeostasis compared to normoxia exposure. Importantly, however, more well-controlled RCTs with detailed metabolic phenotyping (i.e. measurement of tissue-specific insulin sensitivity) are warranted before robust conclusions on the effects of hypoxia exposure on insulin sensitivity and glucose homeostasis can be drawn. In addition, it is important to investigate whether the metabolic effects of hypoxia exposure are age-specific or sex-specific, and depend on the severity, mode (passive hypoxia exposure or hypoxia exposure during exercise) and duration of hypoxia exposure, as well as medication use. Finally, a better understanding of the mechanisms underlying the putative effects of hypoxia on glucose metabolism is needed, since this will contribute to the development and optimization of strategies to prevent and treat impairments in glucose homeostasis and related chronic diseases.

## Abbreviations

AT: Adipose tissue
BMI: Body mass index
HOMA-IR: Homeostatic model assessment for insulin resistance
RCT: Randomized controlled trial
SM: Skeletal muscle
T2DM: Type 2 diabetes
pO_2_: Oxygen partial pressure

## References

[1] Kopelman PG (2000). Obesity as a medical problem. Nature.

[2] Thomas GN, Jiang CQ, Taheri S, Xiao ZH, Tomlinson B, Cheung B (2010). A systematic review of lifestyle modification and glucose intolerance in the prevention of type 2 diabetes. Curr Diabetes Rev.

[3] Alouki K, Delisle H, Bermúdez-Tamayo C, Johri M. Lifestyle interventions to prevent type 2 diabetes: a systematic review of economic evaluation studies. J Diabetes Res. 2016;2016. 10.1155/2016/2159890 PMC4738686 26885527

[4] Howells L, Musaddaq B, McKay AJ, Majeed A. Clinical impact of lifestyle interventions for the prevention of diabetes: an overview of systematic reviews. BMJ open. 2016;6(12). 10.1136/bmjopen-2016-013806 PMC5223710 28003299

[5] Sullivan ED, Joseph DH (1998). Struggling with behavior changes: a special case for clients with diabetes. Diabetes Educ.

[6] Olokoba AB, Obateru OA, Olokoba LB (2012). Type 2 diabetes mellitus: a review of current trends. Oman Med J.

[7] Sparks LM (2017). Exercise training response heterogeneity: physiological and molecular insights. Diabetologia.

[8] Koufakis T, Karras SN, Mustafa OG, Zebekakis P, Kotsa K (2019). The effects of high altitude on glucose homeostasis, metabolic control, and other diabetes-related parameters: from animal studies to real life. High Alt Med Biol.

[9] Stinkens R, Goossens GH, Jocken JW, Blaak EE (2015). Targeting fatty acid metabolism to improve glucose metabolism. Obes Rev.

[10] Lempesis IG, van Meijel RL, Manolopoulos KN, Goossens GH (2020). Oxygenation of adipose tissue: a human perspective. Acta Physiol.

[11] Wood IS, Wang B, Lorente-Cebrián S, Trayhurn P (2007). Hypoxia increases expression of selective facilitative glucose transporters (GLUT) and 2-deoxy-D-glucose uptake in human adipocytes. Biochem Biophys Res Commun.

[12] Yin J, Gao Z, He Q, Zhou D, Guo Z, Ye J (2009). Role of hypoxia in obesity-induced disorders of glucose and lipid metabolism in adipose tissue. American Journal of Physiology-Endocrinology and Metabolism.

[13] Regazzetti C, Peraldi P, Grémeaux T, Najem-Lendom R, Ben-Sahra I, Cormont M (2009). Hypoxia decreases insulin signaling pathways in adipocytes. Diabetes.

[14] Vogel MA, Jocken JW, Sell H, Hoebers N, Essers Y, Rouschop KM (2018). Differences in Upper and Lower Body Adipose Tissue Oxygen Tension Contribute to the Adipose Tissue Phenotype in Humans. J Clin Endocrinol Metab.

[15] Goossens GH, Bizzarri A, Venteclef N, Essers Y, Cleutjens JP, Konings E (2011). Increased adipose tissue oxygen tension in obese compared with lean men is accompanied by insulin resistance, impaired adipose tissue capillarization, and inflammation. Circulation.

[16] Goossens GH, Vogel MA, Vink RG, Mariman EC, van Baak MA, Blaak EE (2018). Adipose tissue oxygenation is associated with insulin sensitivity independently of adiposity in obese men and women. Diabetes Obes Metab.

[17] Vink R, Roumans N, Čajlaković M, Cleutjens J, Boekschoten M, Fazelzadeh P (2017). Diet-induced weight loss decreases adipose tissue oxygen tension with parallel changes in adipose tissue phenotype and insulin sensitivity in overweight humans. Int J Obes.

[18] Duennwald T, Gatterer H, Groop P-H, Burtscher M, Bernardi L (2013). Effects of a single bout of interval hypoxia on cardiorespiratory control and blood glucose in patients with type 2 diabetes. Diabetes Care.

[19] Serebrovska TV, Portnychenko AG, Drevytska TI, Portnichenko VI, Xi L, Egorov E (2017). Intermittent hypoxia training in prediabetes patients: Beneficial effects on glucose homeostasis, hypoxia tolerance and gene expression. Exp Biol Med.

[20] Lecoultre V, Peterson CM, Covington JD, Ebenezer PJ, Frost EA, Schwarz J-M (2013). Ten nights of moderate hypoxia improves insulin sensitivity in obese humans. Diabetes Care.

[21] Mackenzie R, Maxwell N, Castle P, Brickley G, Watt P (2011). Acute hypoxia and exercise improve insulin sensitivity (SI2*) in individuals with type 2 diabetes. Diabetes Metab Res Rev.

[22] Mackenzie R, Elliott B, Maxwell N, Brickley G, Watt P (2012). The effect of hypoxia and work intensity on insulin resistance in type 2 diabetes. J Clin Endocrinol Metab.

[23] Marlatt KL, Greenway FL, Schwab JK, Ravussin E (2020). Two weeks of moderate hypoxia improves glucose tolerance in individuals with type 2 diabetes. Int J Obes.

[24] De Groote E, Britto FA, Bullock L, François M, De Buck C, Nielens H (2018). Hypoxic training improves normoxic glucose tolerance in adolescents with obesity. Med Sci Sports Exerc.

[25] Wiesner S, Haufe S, Engeli S, Mutschler H, Haas U, Luft FC (2010). Influences of normobaric hypoxia training on physical fitness and metabolic risk markers in overweight to obese subjects. Obesity.

[26] Lippl FJ, Neubauer S, Schipfer S, Lichter N, Tufman A, Otto B (2010). Hypobaric hypoxia causes body weight reduction in obese subjects. Obesity.

[27] Morishima T, Hasegawa Y, Sasaki H, Kurihara T, Hamaoka T, Goto K (2015). Effects of different periods of hypoxic training on glucose metabolism and insulin sensitivity. Clin Physiol Funct Imaging.

[28] Morishima T, Mori A, Sasaki H, Goto K (2014). Impact of exercise and moderate hypoxia on glycemic regulation and substrate oxidation pattern. PLoS ONE.

[29] Gutwenger I, Hofer G, Gutwenger AK, Sandri M, Wiedermann CJ (2015). Pilot study on the effects of a 2-week hiking vacation at moderate versus low altitude on plasma parameters of carbohydrate and lipid metabolism in patients with metabolic syndrome. BMC Res Notes.

[30] Chacaroun S, Borowik A, Gonzalez V-EY, Doutreleau S, Wuyam B, Belaidi E, et al. Hypoxic exercise training to improve exercise capacity in obese individuals. Med Sci Sports Exerc. 2020. 10.1249/MSS.0000000000002322 32102058

[31] Klug L, Mähler A, Rakova N, Mai K, Schulz-Menger J, Rahn G (2018). Normobaric hypoxic conditioning in men with metabolic syndrome. Physiol Rep.

[32] Chobanyan-Jürgens K, Scheibe RJ, Potthast AB, Hein M, Smith A, Freund R (2019). Influences of Hypoxia Exercise on Whole-Body Insulin Sensitivity and Oxidative Metabolism in Older Individuals. J Clin Endocrinol Metab.

[33] Shin S, Matsuoka T, So W-Y (2018). Influences of short-term normobaric hypoxic training on metabolic syndrome-related markers in overweight and normal-weight men. J Men's Health.

[34] Brocherie F, Millet GP (2020). Hypoxic exercise as an effective nonpharmacological therapeutic intervention. Exp Mol Med.

[35] Navarrete-Opazo A, Mitchell GS (2014). Therapeutic potential of intermittent hypoxia: a matter of dose. American Journal of Physiology-Regulatory, Integrative and Comparative Physiology.

[36] Trayhurn P (2013). Hypoxia and adipose tissue function and dysfunction in obesity. Physiol Rev.

[37] Görgens SW, Benninghoff T, Eckardt K, Springer C, Chadt A, Melior A (2017). Hypoxia in Combination With Muscle Contraction Improves Insulin Action and Glucose Metabolism in Human Skeletal Muscle via the HIF-1α Pathway. Diabetes.

[38] Siques P, Brito J, Flores K, Ordenes S, Arriaza K, Pena E (2018). Long-term chronic intermittent hypobaric hypoxia induces glucose transporter (GLUT4) translocation through AMP-activated protein kinase (AMPK) in the soleus muscle in lean rats. Front Physiol.

[39] Mackenzie RW, Watt P. A molecular and whole body insight of the mechanisms surrounding glucose disposal and insulin resistance with hypoxic treatment in skeletal muscle. J Diabetes Res. 2016;2016. 10.1155/2016/6934937 PMC4871980 27274997

[40] Fluckey J, Ploug T, Galbo H (1999). Mechanisms associated with hypoxia-and contraction-mediated glucose transport in muscle are fibre-dependent. Acta Physiol Scand.

